# Indications and outcomes of intraocular Lens Exchange among pseudophakic eyes in a Tertiary Referral Center

**DOI:** 10.1186/s12886-023-02871-y

**Published:** 2023-03-28

**Authors:** Mohammadreza Jafarinasab, Masomeh Kalantarion, Sadid Hooshmandi, Kiana Hassanpour, Danial Najdi, Bahareh Kheiri, Hamideh Sabbaghi

**Affiliations:** 1 Department of Ophthalmology, School of Medicine, Shahid Beheshti University of Medical Sciences, Tehran, Iran; 2 Ophthalmic Epidemiology Research Center, Research Institute for Ophthalmology and Vision Science, Shahid Beheshti University of Medical Sciences, 23 Paidar Fard, Bostan 9, Pasdaran Ave, Tehran, 16666 Iran; 3 Department of Medical Education, Virtual School of Medical Education and Management, Shahid Beheshti University of Medical Sciences, Tehran, Iran; 4 Department of Optometry, School of Rehabilitation, Shahid Beheshti University of Medical Sciences, Tehran, Iran; 5 Ophthalmic Research Center, Research Institute for Ophthalmology and Vision Science, Shahid Beheshti University of Medical Sciences, Tehran, Iran

**Keywords:** Indications, Surgical Outcomes, Intraocular Lens Exchange, Pseudophakia

## Abstract

**Purpose:**

To determine the indications and surgical outcomes of intraocular lens (IOL) exchange in pseudophakic patients at Labbafinejad Tertiary Referral Center between 2014 and 2019.

**Methods:**

In this retrospective interventional case series, the medical records of 193 patients with a history of IOL exchange were reviewed. Preoperative data, including clinical characteristics, indications of the first and second IOL implantation, intra- and postoperative complications due to IOL exchange, and the pre-and postoperative refractive error and best-corrected visual acuity (BCVA) were considered the outcome measures in this study. All postoperative data were analyzed at least six months after follow-up.

**Results:**

The mean age of our participants was 59.13 ± 20.97 years old at the time of the IOL exchange, with a male percentage of 63.2%. The mean follow-up after the IOL exchange was 15.72 ± 16.28 months. The main indications of IOL exchange were IOL decentration (50.3%), corneal decompensation (30.6%), and residual refractive errors (8.3%). 57.10% of patients with the postoperative spherical equivalent at -2.00 diopter (D) to + 2.00D. The mean best-corrected visual acuity was 0.82 ± 0.76 LogMAR before the IOL exchange and was improved to 0.73 ± 0.79 LogMAR after the surgery. Corneal decompensation (6.2%), glaucoma (4.7%), retinal detachment (4.1%), cystoid macular edema (2.1%), and uveitis (1%) were found as the postoperative complications. There was only one case with suprachoroidal hemorrhage during IOL exchange.

**Conclusions:**

IOL decentration followed by corneal decompensation was the most common indication of IOL exchange. After IOL exchange, the most complications during follow-up were corneal decompensation, glaucoma, retinal detachment, and cystoid macular edema.

## Introduction

Cataract disease is the second cause of visual impairment worldwide [1, 2]. Cataract surgery is the most common intraocular surgical procedure promoting the quality of life and vision [3]. Primary and secondary implantation of intraocular lenses (IOLs) in cataract surgery affect the postoperative outcomes [4].

Although this type of intraocular surgery is safe and primarily performed without any severe complications, the second operation may be necessary for some patients to remove or exchange the IOL [5–7]. Bothun et al. demonstrated the increasing rate of IOL exchange in pseudophakic patients over the last 30 years in a population-based study [4].

Various causes are reported as indications of IOL exchange regarding the IOL design and its biomaterial [8]. In a review article conducted by Fernández-Buenaga et al. [7]. IOL decentration, inaccurate IOL power calculation, and IOL opacification were reported as the three most common indications for IOL explanation. Regarding the type of IOL, Marques et al. [9] found that intraocular inflammation and IOL decentration were the primary indications in eyes implanted by anterior and posterior chamber IOLs, respectively. Furthermore, refractory cystoid macular edema, pseudophakic bullous keratopathy, and patient dissatisfaction have been reported as the other indications in various studies [6, 10–12]. Intraoperative complications and preoperative ocular comorbidity are also associated with causes of IOL exchange.

Recently, clinical outcomes of the IOL exchange are also evolving. Therefore, optimal visual results with fewer complications are expected after the IOL exchange [6, 7]. Recognition of the factors leading to IOL exchange and the final clinical outcomes after the procedure helps more accurate decision-making in similar conditions. The present study was designed to determine the indications and clinical outcomes of IOL exchange in pseudophakic patients operated on in a Tertiary Referral Center.

## Methods

Medical records of 193 pseudophakic patients who underwent IOL exchange were retrospectively reviewed. All patients were operated on at Labbafinejad Medical Center, Tertiary Referral Center, Tehran, Iran, between 2014 and 2019.

Demographic characteristics and clinical data including any systemic diseases, ocular comorbidity, history of previous ocular surgery, type, material, design, and power of the first and the second IOLs, intraocular site of the implanted IOL, intraocular pressure (IOP), indications for IOL explantation, intra- and postoperative complications due to the IOL exchange, duration between the first and the second IOL implantations were recorded. Furthermore, the pre-and postoperative refractive error and the best-corrected visual acuity (BCVA), were noted. All postoperative data were recorded for at least six months after the second IOL implantation.

The IOL power was calculated using an IOLMaster optical biometer (V.2.02, Carl Zeiss Meditec) based on the SRK/T formula in eyes with normal and long axial length (AL) (> 22 mm) and Hoffer Q in eyes with AL < 22 mm At first, all patients underwent a comprehensive ophthalmic examination including assessment of PGVA and BCVA by Snellen E-chart at a distance of 6 m under the daylight illumination, measurement of refractive error using an auto refractometer (RM-8800; Topcon Medical, Oakland, NJ, USA) or retinoscope (HEINE BETA®200; Germany) if auto refractometer was not possible. Intraocular pressure was also measured by Goldmann applanation tonometry. Anterior ocular segment examination was performed, and a fundus examination was conducted through dilated pupils.

### Surgical technique

Two experienced surgeons performed the surgeries (MRJ, AF). After the preparation and draping of the patients and providing anesthesia, the wound was opened. In eyes with PCIOL, the adhesions of the IOLs to the capsular bag were gently released using an ophthalmic viscoelastic device (OVD). If the separation of the IOL was not possible due to the fibrotic adhesions, the haptics of the IOLs was cut and left in place. In eyes with ACIOL, the IOL was extracted from a 6 mm incision. To reduce the endothelial damage, the haptics were cut, and the extraction was done in two stages in patients with severe adhesions of the haptics to the iris.

The IOL was removed from the corneal wound with extra caution to the endothelium. An ophthalmic viscoelastic device (OVD) was used to the posterior chamber IOLs (MA60AC Acrysof, Alcon) with scleral fixation or AC IOL (Artisan, Ophtec, Groningen, Netherlands) were placed in patients on an individual basis. The 3-piece IOL was placed in the ciliary sulcus in patients with adequate capsular support. The ciliary sulcus IOL was sutured to the iris or sclera to secure the IOL’s stability based on the surgeon’s decision. In patients with inadequate capsular support iris-claw, AC IOL (Artisan, Ophtec, Groningen, Netherlands) was placed. At the time of the study, retropupillary fixation of Artisan was not a preferred method by the authors. The wound was sutured with nylon 10 − 0.

The postoperative regimen included topical antibiotic QID for one week and frequent topical steroid tapering over 1.5 months.

### Main outcome measures

Indication of IOL exchange was considered the primary outcome measure. In addition, BCVA, refractive error, and postoperative complications were analyzed as secondary outcomes.

### Statistical analysis

Mean, standard deviation, and percentage were used to describe the data. SPSS version 25.0 was used to analyze the data. A p-value less than 0.05 was considered statistically significant. The indications and outcomes between the posterior and anterior chamber IOLs were compared using the chi-square test.

## Results

In the present study, a total of 193 pseudophakic patients with an average age of 59.13 ± 20.9 years old at the time of IOL exchange and a male percent of 63.2% were included (Table 1).

**Table 1:** Demographic characteristics of the study subjects

Variables		PC IOL	AC IOL	P value
Age	Mean ± SD	59.7 ± 20.3	57.1 ± 22.7	0.81*
	Median (Range)	65 (20 to 85)	62.5 (18 to 90)	
Sex	Female	46 (35.9%)	25 (38.5%)	0.04**
	Male	82 (64.1%)	40 (61.5%)	
Eye	Right	60 (46.9%)	35 (53.8%)	0.035**
	Left	68 (53.1%)	30 (46.2%)	
Follow-up	Mean ± SD	16.7 ± 18.6	13.9 ± 10.6	0.76*
	Median	12 (1 to 84)	12 (1 to 36)	
Ocular Comorbidities	PEX	31 (24.2)	9 (13.8)	0.05^#^
	Glaucoma	4 (3.1%)	2 (3.1%)	
	Retinal Pathology	8 (6.3%)	2 (3.1%)	
	MMP	1 (0.7%)	0	
Systemic conditions	Marfan Syndrome	1 (0.5%)	0	0.76^#^
	HTN	11 (8.6%)	12 (18.5%)	
	DM	13 (10.2%)	11 (16.9%)	
	IHD	3 (2.3%)	2 (3.1%)	

**Fig. 1:**
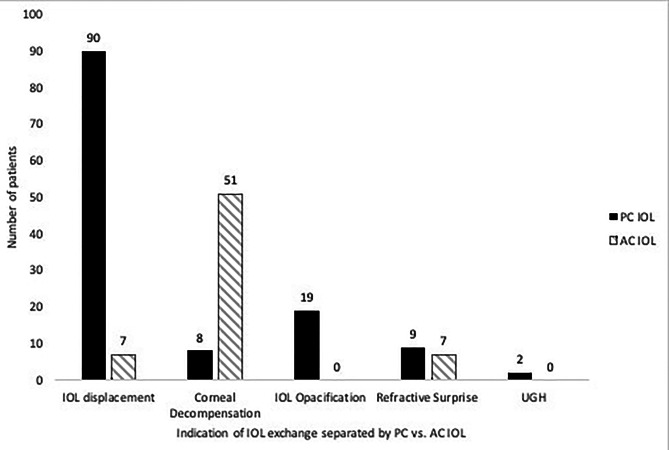
The Indications of IOL exchange separated by type of explanted IOL. IOL: intraocular lens; AC: anterior chamber; PC: posterior chamber; UGH: Uveitis-Glaucoma-Hyphema.

*Based on T-test; Based on Chi-square; ^#^ Based on fisher exact test. PEX: Pseudoexfoliation Syndrome; HTN: Hypertension; DM: Diabetes Mellitus; IHD: Ischemic Heart Disease; MMP: Mucous membrane pemphigoid; SD: Standard Deviation;

The main indications for IOL exchange were IOL displacement in 97 eyes (50.3%), corneal decompensation in 59 eyes (30.6%), and residual refractive errors in 16 eyes (8.3%). The other causes include IOL opacification in 19 eyes (9.8%) and Uveitis-Glaucoma-Hyphema syndrome in 2 eyes (1%). (Table 2) Concomitant surgical procedures were penetrating keratoplasty in 21 patients (10.9%) and Descemet stripping automated endothelial keratoplasty (DSAEK) in 30 (15.5%.). The indication for keratoplasty was clinical corneal edema at the time of the IOL exchange. The patients with low endothelial cell count without frank corneal edema were informed of the need for sequential keratoplasty (Fig. 1).

**Table 2:** Indications and Interval of IOL exchange for study participants

Factors	PC IOL Number (Percent)	AC IOL	Time (Mean ± SD)
IOL dislocation	90 ( 70.3%)	7 (10.8%)	4.05 ± 5.1
Corneal Decompensation	8 (6.3%)	51 (78.5%)	8.14 ± 6.9
Refractive Surprise	9 (7%)	7 (10.7%)	0.87 ± 0.6
IOL Opacification	19 ( 14.8%)	0 ( 0%)	5.91 ± 5.2
UGH	2 (1.6%)	0 (0%)	0.63 ± 0.1

IOL: intraocular lens; UGH: Uveitis-Glaucoma-Hyphema; SD: standard deviation

The average time between the initial surgery and the IOL exchange was 4.12 ± 5.6 years (Median 2 years, range one month to 32 years). The time interval between the initial surgery and IOL exchange was 0.57 ± 0.9 years and 4.05 ± 5.1 years for out of the bag and in the bag IOL dislocation, respectively. While this period was 8.14 ± 6.9 years in patients with corneal decompensation.

The explanted IOLs consisted of PC IOLs in 128 eyes (66.3%) and AC IOLs in 65 (33.7%). The indications for exchange of PC IOL were IOL displacement in 90 (70.3%), corneal decompensation in 8 (6.3%), opacification in 19 (14.8%), refractive surprise in 9 (7%), and UGH in 2 (1.6%). The indications for AC IOL exchange were corneal decompensation in 51 (78.5%) IOL malposition in 7 (10.8%), and refractive surprise in 7 (10.7%) of patients (Table 3).

**Table 3:** Types of explanted IOL.

IOL Type	Total		
AC IOL	65 (33.7%)	Angel supported	55 (84.6%)
		Iris-claw	10 (15.4%)
PC IOL	128 (66.3%)	Three-piece	114 (89.1%)
		One-piece	10 (5.2%)
		PMMA	4 (2.1%)

IOL: intraocular lens; AC: anterior chamber; PC: posterior chamber; PMMA: polymethyl methacrylate

The secondary implanted IOL were scleral-fixated in 14 (7.2%), iris-fixated in 11 (5.7%), iris claw fixated IOL in 142 (73.6%), and in the ciliary sulcus IOL in 11 (5.7%) patients. Fifteen (7.8%) patients remained aphakic (Table 4).

**Table 4:** Type of implanted IOL and postoperative outcome

Factors		Number (percent)
IOL implantation		
	In the bag	11 (5.7%)
	In ciliary sulcus (Iris fixation)	11 (5.7%)
	In ciliary sulcus (Scleral fixation)	14 (7.2%)
	Iris-Claw (Artisan)	142 (73.6%)
	Aphakia	15 (7.8%)
Complications	Glaucoma	9 (4.7%)
	Cystoid Macular Edema	4 (2.1%)
	Retinal Detachment	8 (4.1%)
	Corneal Decompensation	12 (6.2%)
	Uveitis	2 (1%)

IOL: intraocular lens

The average follow-up time after IOL exchange was 15.72 ± 16.28 months. There were 57.10% cases with the postoperative spherical equivalent at the range of -2.00 diopter (D) to + 2.00D. Preoperative BCVAwas 0.82 ± 0.76 LogMAR reached 0.73 ± 0.79 LogMAR (P = 0.045).

In subgroup analysis, preoperative BCVA was comparable between patients with PC IOL and AC IOL. The average BCVA was 0.70 ± 0.61 in the group with IOL placed in the posterior chamber (Iris-fixated, scleral fixated and in the bag) while the average BCVA was 0.83 ± 0.81 in patients with Iris claw fixation IOLs. There was no significant difference between the two groups in terms of BCVA. (P = 0.054)

The average spherical equivalent was _ 2.6 ± 6.3 preoperatively which significantly reduced to _ 1.7 ± 4.2 postoperatively. (P = 0.023) In subgroup analysis, the average SE was significantly more negative in patients with iris-claw fixated IOL compared to the patients who underwent posterior chamber IOL. ( _2.8 vs. _1.2, P = 0.032)

In patients undergoing concomitant penetrating keratoplasty and DSAEK, the preoperative BCVA was 1.34 ± 0.6 which significantly improved to 0.82 ± 0.78 postoperatively. (P = 0.031)

The average SE in this group was not reliable preoperatively. Postoperative SE at the last follow-up was _ 4.00 ± 3.5 and + 1.8 ± 2.5 in patients with successful PKP and DSAEK, respectively.

Corneal decompensation 12 (6.2%), glaucoma 9 (4.7%), retinal detachment 8 (4.1%), cystoid macular edema 4 (2.1%), and uveitis 2 (1.0%) were found as the postoperative complications. Only one patient experienced the suprachoroidal hemorrhage during IOL exchange. No endophthalmitis was observed.

## Discussion

The results of the present study demonstrate that the main indications for IOL exchange were IOL displacement (subluxation or decentration) followed by corneal decompensation in seven years at a tertiary eye center. Over the last decades, indications for IOL exchange have changed. Refractive surprise, corneal decompensation, and IOL opacification were more common in the early 20s, while IOL dislocation became more prevalent later. The main studies reporting the clinical outcome of IOL exchange are summarized in Table 5 [13–19].

**Table 5:** Review of the studies reporting the clinical outcome of IOL exchange

Author (Year)	No. of eyes	Mean follow up	Indications for exchange	Visual Outcome	Complications
Katarakt et al. 2021[13]	127	34.9 m	Dislocation, corneal edema	Mean BCVA improved	CME
Rojas et al.2020[25]	141	11.19 m	dislocation, corneal decompensation	Mean BCVA significantly increased	CME glaucoma
Goemaere et al. 2020[27]	492		Opacification, dislocation	Mean BCVA significantly increased	Glaucoma CME
Bothun et al. 2018 [4]	80		dislocation, corneal edema	Not reported	dislocation, corneal edema
Buenaga et al. 2017[7]	257		Dislocation, incorrect IOL power	significant improvement in (BCVA)	intraocular pressure increase
Chai et al. 2017[14]	69		Dislocation, retinal detachment	72.5% of patients improved	Not reported
Davies et al. 2016[15]	109	6 m	Dislocation, dissatisfaction	78.9% of cases improved	PCO CME
Chan et al. 2015[16]	98	28.3 m	Dislocation, UGH	All eyes improved	Glaucoma Uveitis
Oltulu et al. 2015[18]	93		bullous keratopathy AC IOL, and dislocation in PC IOL	Improved in the PCIOL group. But did not improve in the ACIOL group.	Bullous keratopathy
Jones et al. 2014[6]	57	14.9 m	dislocation, incorrect IOL power, patient dissatisfaction	88% of all eyes were 20/40 or better	PCO
Leysen et al. 2009[8]	113	7.55 m	opacification, decentration, dislocation, capsule phimosis	improved in all cases	Glaucoma
Jiraskova et al. 2007[17]	23		Opacification, malposition	no significant difference	Glaucoma, hypotony
Marques et al. 2006[9]	49	35.5 m	inflammation in AC IOL and dislocation in PC IOL	improved by 80%	CME
Jin et al. 2005[10]	51	22 m	Incorrect IOL power, decentration, and glare	90.2% of patients obtained 20/40 or better	CME, uveitis, PCO
Sinskey et al. 1993[19]	79		dislocation, endothelial decompensation	72% had better visual acuity	retinal detachment, glaucoma, corneal decompensation
Lyle et al. 1992[11]	101	23 m	Bullous keratopathy, Lens dislocation, incorrect IOL power	88% having two lines of improvement	CME, hyphema, glaucoma, and PCO

BCVA: best corrected visual acuity. CME: cystoid macular edema. PCO: posterior capsule opacification. IOL: intraocular lens. AC: anterior chamber. PC: posterior chamber

IOL displacement remains a main indication for IOL exchange. IOL displacement of PCIOL is divided into two main categories “in the bag” when the capsule lens complex is displaced and out of the bag, when occurs due to sulcus placement of the IOL. IOL displacement risk is estimated at 0.1% at ten years and 1.7% at 25 years [20]. Various factors including intraoperative complications, ocular factors like PEX, RP, long axial length, and history of previous vitreoretinal surgery or trauma cause IOL displacement. In patients with PEX, progressive separation of the zonules or higher risk of intraoperative complications like posterior capsular rupture (PCR). in a study by Jones et al.,. [6] 40% of PEX patients with IOL dislocation had a PCR intraoperatively. Capsular shrinkage and zonular dehiscence are the proposed mechanisms in retinitis pigmentosa [21].

Corneal decompensation was the second most common indication for IOL exchange in the whole cohort of our patients and the leading cause in patients with AC IOL. This result is in line with studies reporting the indications of IOL exchange in patients with AC IOL. Duran et al. [22] evaluated the indications for and outcomes of 29 cases undergoing anterior chamber IOL explantation and reported that corneal decompensation was the indication for IOL explantation in 22 cases. Three underwent keratoplasty and scleral-fixated IOL implantation; no surgical intervention was performed in the remaining cases. In our center, corneal decompensation was detected by specular microscopy and clinical examination. Concomitant DSAEK was performed in patients with clinical edema while IOL explantation was performed when the endothelial cell counts showed a critical decrease.

Despite the absence in our series, one emerging indication of IOL exchange is patients’ dissatisfaction after multifocal IOL implantation [23, 24] We believe that the future trend in our center will also change toward a higher proportion of patients with multifocal IOL exchange as we can see after 2019 in our center.

IOL explantation can be performed from the anterior or pars plana approach. All patients were explanted through the anterior approach in our series because the patients needing concomitant VR surgery were excluded from this study. In a review by De Rajos et al. [25] the surgical approach for IOL explantation was anterior in 104 cases (73.75%) and posterior in 37 (26.24%). Vitrectomy was performed concurrently with IOL removal in 135 cases, from a limbal approach in 98 cases, and pars plana vitrectomy in 37 cases. The two groups were comparable in terms of visual outcome and postoperative complications.

The time interval between the first surgery and IOL explantation was 4.12 ± 5.6 years in our series, which is comparable with most studies. De Rajos et al. [25] reported the average time from original surgery to IOL explantation was 7.89 ± 5.81 years (range 0.08 to 29); 9.31 ± 7.54 years (range 0.75 to 29) for anterior chamber IOLs, and 7.70 ± 5.55 years (range 0.08 to 28.25) for posterior chamber IOLs (p = 0.529) There was no significant difference between the in-the-bag, the out-of-the-bag IOL dislocation group. In the study by Vounotrypidis et al. [26] the mean period between the primary surgery and the secondary IOL implantation was 8.4 ± 6.5 years (range 0 − 32 years). Goemaere et al. [27] reported that the shortest time interval is in refractive error (29.42 ± 42.46 months), and the most prolonged time is for corneal decompensation (151.83 ± 111.07 months).

There are various surgical options to implant the secondary IOL, including anterior iris-claw IOLs, scleral or iris-fixated IOLs, and retropupillary fixation of iris-claw IOLs. The choice of IOL depends on the availability of IOLs, the status of the posterior capsule and iris, and the surgeon’s experience and preference. In a report by De Rajos et al. [25] retropupillary iris-claw IOL was used in most patients with a favorable outcome. While angle-supported IOLs were implanted in half of the patients in the study by Vounotrypidis and colleagues [26] Iris claw AC-IOL was the most commonly used secondary IOL in our series, followed by scleral-fixated PCIOL. In an ophthalmic technology assessment, reported by the American academy of ophthalmology in 2020, various techniques were compared when there is no capsular support. The OTA team concluded that any single IOL implantation technique in the absence of zonular support showed superiority. Moreover, iris-claw fixated IOL like Artisan was discussed as a good option despite no approval by FDA [28]. Our results add to the literature that iris-claw IOLs can be used with a favorable safety profile after IOL exchange.

Various factors affect the visual outcome after IOL exchange. Postoperative astigmatism due to the lens tilt and incidence of postoperative complications, including IOP rise, RD, or corneal decompensation, limit visual acuity improvement after IOL explantation. The high rate of corneal decompensation could be attributed to the present protocol of our center, in which patients with the clear cornea and abnormal endothelial cell counts did not undergo concomitant keratoplasty.

The retrospective nature of the disease limits our study. Incomplete data in some patients, surgery by different surgeons, and loss of follow-up in some patients leading to attrition bias are among the other limitations of the present study. Patients that left aphakic consist of a high proportion of our patients. These patients were generally patients without capsular support and with early evidence of clinical corneal decompensation and iris claw placement was controversial. Therefore, we decided to leave them aphakic. However, the present study reports a large number of patients from a tertiary referral center with an extended follow-up.

In conclusion, IOLs implantation may require further surgical intervention to prevent more damage to the eye or restoration of visual function. This intervention’s causes are different regarding population characteristics, type of IOL (PC vs. AC), and the study time. In the current research, complications of previous surgery lead to the displacement of the IOL in PC IOL while corneal decompensation in AC IOL was the most common cause for secondary surgical procedures. The main side effects of the second surgery in the current study were glaucoma, CME, RRD, and corneal decompensation.

## Data Availability

The datasets used and/or analyzed during the current study available from the corresponding author on reasonable request.

## References

[1] Gilbert C, Foster A (2001). Childhood blindness in the context of VISION 2020: the right to sight. Bull World Health Organ.

[2] Shahdadi H (2018). Frequency of cataract in Iran: a meta-analysis and systematic review. Middle East African journal of ophthalmology.

[3] Behndig A (2011). One million cataract surgeries: swedish national cataract register 1992–2009. J Cataract Refractive Surg.

[4] Bothun ED (2018). Population-based incidence of intraocular lens exchange in Olmsted County, Minnesota. Am J Ophthalmol.

[5] Goh ES. *Maximising safety of cataract surgery training: improving patient safety by reducing cataract surgery complication rates*. International journal of health care quality assurance, 2009. 10.1108/09526860910975634 19725373

[6] Jones JJ, Jones YJ, Jin GJ (2014). Indications and outcomes of intraocular lens exchange during a recent 5-year period. Am J Ophthalmol.

[7] Fernández-Buenaga R, Alió JL (2017). Intraocular lens explantation after cataract surgery: indications, results, and explantation techniques. Asia-Pacific J Ophthalmol.

[8] Leysen I (2009). Surgical outcomes of intraocular lens exchange: five-year study. J Cataract Refractive Surg.

[9] Marques FF (2007). Longitudinal study of intraocular lens exchange. J Cataract Refractive Surg.

[10] Jin GJ, Crandall AS, Jones JJ (2005). Changing indications for and improving outcomes of intraocular lens exchange. Am J Ophthalmol.

[11] Lyle WA, Jin J-C (1992). An analysis of intraocular lens exchange.

[12] Mamalis N (2008). Complications of foldable intraocular lenses requiring explantation or secondary intervention—2007 survey update. J Cataract Refractive Surg.

[13] OZULKEN K, Cubuk MO, Yuksel E. Effects of Static Cyclotorsion compensation of refractive outcomes and level of Vision in Excimer laser surgery. Volume 16. Glokom-Katarakt/Journal of Glaucoma-Cataract; 2021. 1.

[14] Chai F (2017). A pilot study of intraocular lens explantation in 69 eyes in chinese patients. Int J Ophthalmol.

[15] Davies EC, Pineda R (2016). Intraocular lens exchange surgery at a tertiary referral center: indications, complications, and visual outcomes. J Cataract Refractive Surg.

[16] Chan TC (2015). Intraocular lens explantation in chinese patients: different patterns and different responses. Int Ophthalmol.

[17] Jirásková N, Rozsíval P, Kohout A (2007). A survey of intraocular lens explantation: a retrospective analysis of 23 IOLs explanted during 2005. Eur J Ophthalmol.

[18] Oltulu R (2015). Intraocular lens explantation or exchange: indications, postoperative interventions, and outcomes. Arquivos brasileiros de oftalmologia.

[19] Sinskey RM, Amin P, Stoppel JO (1993). Indications for and results of a large series of intraocular lens exchanges. J Cataract Refractive Surg.

[20] Pueringer SL, Hodge DO, Erie JC (2011). Risk of late intraocular lens dislocation after cataract surgery, 1980–2009: a population-based study. Am J Ophthalmol.

[21] Hayashi K (1998). Anterior capsule contraction and intraocular lens dislocation after implant surgery in eyes with retinitis pigmentosa. Ophthalmology.

[22] DURAN S et al. *Ön Segment Göz İçi Mercek Çıkarımı Sebepleri ve Sonuçları*.Glokom-Katarakt/Journal of Glaucoma-Cataract, 2013. 8(3).

[23] Al-Shymali O (2022). Multifocal intraocular lens exchange to monofocal for the management of neuroadaptation failure. Eye and Vision.

[24] Galor A (2009). Intraocular lens exchange surgery in dissatisfied patients with refractive intraocular lenses. J Cataract Refractive Surg.

[25] de Rojas M (2020). Intraocular lens explantation in Spain: indications and outcomes at a tertiary referral center from 2010 to 2018. Int Ophthalmol.

[26] Vounotrypidis E (2019). Secondary intraocular lens implantation: a large retrospective analysis. Graefe’s Archive for Clinical and Experimental Ophthalmology.

[27] Goemaere J (2020). Fifteen years of IOL exchange: indications, outcomes, and complications. J Cataract Refractive Surg.

[28] Shen JF (2020). Intraocular lens implantation in the absence of zonular support: an outcomes and safety update: a report by the American Academy of Ophthalmology. Ophthalmology.

